# Analysis of patients with diabetes and complicated intra-abdominal infection or complicated urinary tract infection in phase 3 trials of ceftolozane/tazobactam

**DOI:** 10.1186/s12879-017-2414-9

**Published:** 2017-05-02

**Authors:** Myra W. Popejoy, Jianmin Long, Jennifer A. Huntington

**Affiliations:** Merck & Co., Inc., 2000 Galloping Hill Road, Kenilworth, NJ 07033 USA

**Keywords:** Ceftolozane/tazobactam, Complicated urinary tract infections, Complicated intra-abdominal infections, Diabetes mellitus

## Abstract

**Background:**

Diabetes mellitus and hyperglycemia are associated with increased susceptibility to bacterial infections and poor treatment outcomes. This post hoc evaluation of the treatment of complicated intra-abdominal infections (cIAI) and complicated urinary tract infections (cUTI) aimed to evaluate baseline characteristics, efficacy, and safety in patients with and without diabetes treated with ceftolozane/tazobactam and comparators. Ceftolozane/tazobactam is an antibacterial with potent activity against Gram-negative pathogens and is approved for the treatment of cIAI (with metronidazole) and cUTI (including pyelonephritis).

**Methods:**

Patients from the phase 3 ASPECT studies with (*n* = 245) and without (*n* = 1802) diabetes were compared to evaluate the baseline characteristics, efficacy, and safety of ceftolozane/tazobactam and active comparators.

**Results:**

Significantly more patients with than without diabetes were 65 years of age or older; patients with diabetes were also more likely to weigh ≥75 kg at baseline (57.1% vs 44.5%), to have renal impairment (48.5% vs 30.2%), or to have APACHE II scores ≥10 (33.8% vs 17.0%). More patients with diabetes had comorbidities and an increased incidence of complicating factors in both cIAI and cUTI. Clinical cIAI and composite cure cUTI rates across study treatments were lower in patients with than without diabetes (cIAI, 75.4% vs 86.1%, *P* = 0.0196; cUTI, 62.4% vs 74.7%, *P* = 0.1299) but were generally similar between the ceftolozane/tazobactam and active comparator treatment groups. However, significantly higher composite cure rates were reported with ceftolozane/tazobactam than with levofloxacin in patients without diabetes with cUTI (79.5% vs 69.9%; *P* = 0.0048). Significantly higher rates of adverse events observed in patients with diabetes were likely due to comorbidities because treatment-related adverse events were similar between groups.

**Conclusions:**

In this post hoc analysis, patients with diabetes in general were older, heavier, and had a greater number of complicating comorbidities. Patients with diabetes had lower cure rates and a significantly higher frequency of adverse events than patients without diabetes, likely because of the higher rates of medical complications in this subgroup. Ceftolozane/tazobactam was shown to be at least as effective as comparators in treating cUTI and cIAI in this population.

**Trial registration:**

cIAI, NCT01445665 and NCT01445678 (both trials registered prospectively on September 26, 2011); cUTI, NCT01345929 and NCT01345955 (both trials registered prospectively on April 28, 2011).

## Background

In recent decades, the incidence and prevalence of diabetes have increased rapidly [1], with recent studies estimating that 422 million people worldwide are affected [2]. In addition to the burden directly imposed by the condition, patients with diabetes mellitus and hyperglycemia have been shown to have increased susceptibility to bacterial infections and poor outcomes, including increased risk for hospitalization, reduced cure rates, and increased mortality due to infection [3, 4]. Furthermore, patients with diabetes commonly have comorbidities that may further affect their response to treatment; for example, both cardiovascular disease and chronic kidney disease appear to be predictors of lengthened hospital stay and infection-related mortality [5–7].

Ceftolozane/tazobactam, an antibacterial with potent activity against Gram-negative pathogens [8, 9], is approved by the US Food and Drug Administration and the European Medicines Agency for the treatment of patients with complicated intra-abdominal infections (cIAI) when used in combination with metronidazole and for the treatment of patients with complicated urinary tract infections (cUTI), including pyelonephritis [10, 11]. Ceftolozane/tazobactam was studied in a large phase 3 clinical trial program (Assessment of the Safety Profile and Efficacy of Ceftolozane/Tazobactam [ASPECT]) in patients with cIAI or cUTI. In ASPECT-cIAI (NCT01445665 and NCT01445678), ceftolozane/tazobactam plus metronidazole was noninferior to meropenem in patients with cIAI [12]. In ASPECT-cUTI (NCT01345929 and NCT01345955), ceftolozane/tazobactam demonstrated efficacy superior to that of high-dose levofloxacin in patients with cUTI [13].

Herein we present a post hoc investigation of baseline characteristics, efficacy, and safety from patients with or without a reported medical history of diabetes in the phase 3 ASPECT trials. The aims of this evaluation were to examine the baseline characteristics of patients with and without diabetes who were enrolled in the ASPECT trials and to assess whether ceftolozane/tazobactam was safe and effective in treating cIAI and cUTI in patients with diabetes.

## Methods

### Study design

Two multicenter, multinational, randomized (1:1 ratio), double-blind, noninferiority trials were conducted from 2011 to 2013 (Merck protocols: CXA-cIAI-10-08, CXA-cIAI-10-09, CXA-cUTI-10-04, and CXA-cUTI-10-05). Studies were conducted in accordance with the principles of Good Clinical Practice and were approved by the appropriate institutional review boards and regulatory agencies [12, 13]. In ASPECT-cIAI, adults with cIAI in need of surgical intervention were assigned to receive intravenous (IV) ceftolozane/tazobactam 1.5 g plus metronidazole 500 mg every 8 h (q8h) or IV meropenem 1 g plus placebo q8h for 4 to 14 days. In ASPECT-cUTI, adults with cUTI (including pyelonephritis) were assigned to receive IV ceftolozane/tazobactam 1.5 g q8h or IV levofloxacin 750 mg/day for 7 days.

### Patients

Patients enrolled in the trials were classified into subgroups with and without diabetes, based on their reported medical history, and all analyses were evaluated between these two subgroups. Baseline demographics and characteristics were recorded descriptively. Between-group differences were determined, and statistical significance was calculated using the Miettinen and Nurminen method [14].

### Efficacy assessments

In ASPECT-cIAI, clinical cure, defined as complete resolution or significant improvement in signs and symptoms of index infection with no additional antibiotics or surgical intervention, was assessed at the test-of-cure (TOC) visit (24–32 days after study drug start). In ASPECT-cUTI, composite cure, defined as both clinical cure (complete resolution or significant improvement in all signs and symptoms) and microbiologic eradication (reduction in all baseline uropathogens to <10^4^ CFU/mL in urine culture) was assessed at the TOC visit (5–9 days after the end of therapy). For this analysis, clinical cure and composite cure rates were compared between patients with and without diabetes, with indeterminate responses imputed as clinical failures, and Wilson score intervals were used to calculate confidence intervals.

### Safety assessments

Safety and tolerability were assessed by recording adverse events (AEs). AEs were categorized by the investigator as treatment related (possibly, probably, or definitely) or not treatment related. Data were recorded descriptively. Between-group differences were determined, and statistical significance was calculated using the Miettinen and Nurminen method [14].

### Analysis populations

The cIAI microbiologic intention-to-treat population included all randomly assigned patients with cIAI with ≥1 baseline intra-abdominal pathogen regardless of receipt of, or susceptibility to, study drug. The cUTI microbiologic modified intention-to-treat population included all randomly assigned patients with cUTI with ≥1 dose of study drug and ≥1 uropathogen at baseline, regardless of susceptibility to study drug. The integrated safety population included all patients with cIAI or cUTI who received any amount of study drug.

## Results

### Patient population and disposition

The pooled analysis population comprised 979 patients from ASPECT-cIAI and 1068 patients from ASPECT-cUTI [12, 13], including 245 patients with diabetes and 1802 without diabetes. Patient disposition is shown in Table 1. Patient groups (with diabetes and without diabetes) had similar rates of study completion and study drug completion and similar reasons for discontinuation. In ASPECT-cUTI, negative/contaminated urine culture (12.1% and 18.8%, respectively) was the most common reason for early discontinuation of study drug, which was required per protocol.

**Table 1:** Patient disposition (safety population)

Disposition, n (%)	Diabetes *n* = 245	No diabetes *n* = 1802
Patients completing the studies	230 (93.9)	1726 (95.8)
Most common reasons for premature withdrawal from the study		
AEs	5 (2.0)	16 (0.9)
Patient’s decision	6 (2.4)	29 (1.4)
Patients completing study drug	200 (81.6)	1528 (84.8)
Most common reasons for discontinuing study drug		
AEs	8 (3.3)	32 (1.8)
Patient’s decision	7 (2.8)	36 (2.0)
Lack of efficacy	5 (2.0)	11 (0.6)

*AE* adverse event

### Baseline characteristics

Baseline demographics and disease characteristics are reported in Table 2. In the subgroups with and without diabetes, most patients were white, and slightly more women than men were included. Patients were evenly distributed between treatment arms in the subgroups with and without diabetes (data not shown).

**Table 2:** Patient demographics and disease characteristics at baseline (MITT/cIAI population and mMITT/cUTI population)

Parameter	Diabetes *n* = 198	No diabetes *n* = 1408	Difference^a^; *P* value
cIAI, *n* (%)	65 (32.8)	741 (52.6)	—
cUTI, *n* (%)	133 (67.2)	667 (47.4)	—
Sex, *n* (%)			
Male	73 (36.9)	601 (42.7)	5.8; 0.12061
Female	125 (63.1)	807 (57.3)	−5.8; 0.12061
Age, years			
Mean (SD)	60 (13.9)	48 (18.9)	—
≥ 18–<65, *n* (%)	123 (62.1)	1099 (78.1)	15.9; <0.00001
≥ 65–<75, *n* (%)	46 (23.2)	166 (11.8)	−11.4; <0.00001
> 75, n (%)	29 (14.6)	143 (10.2)	−4.5; 0.05581
Race, *n* (%)			
White	159 (80.3)	1282 (91.1)	10.7; <0.00001
Black	0 (0.0)	17 (1.2)	1.2; 0.12020
Asian	30 (15.2)	64 (4.5)	−10.6; <0.00001
Other	9 (4.5)	45 (3.2)	−1.4; 0.29496
Geographic region, *n* (%)			
North America	17 (8.6)	59 (4.2)	−4.4; 0.00640
South America	26 (13.1)	127 (9.0)	−4.1; 0.06511
Western Europe	3 (1.5)	27 (1.9)	0.4; 0.69541
Eastern Europe	118 (59.6)	1095 (77.8)	18.2; <0.00001
Rest of world	34 (17.2)	100 (7.1)	−10.1; <0.00001
Weight, kg			
Mean (SD)	79 (17.2)	74 (17.2)	−5.28; 0.00003
≥ 75 kg, *n* (%)	113 (57.1)	627 (44.5)	−12.5; 0.00092
BMI, kg/m^2^, mean (SD)	29 (5.8)	26 (5.4)	−3.24; <0.00001
APACHE II score (cIAI), *N* ^b^	65	740	
< 10, *n* (%)	43 (66.2)	614 (83.0)	−16.8; <0.0008
≥ 10, *n* (%)	22 (33.8)	126 (17.0)	
Baseline creatinine clearance, *n* (%)			
Missing	1 (0.5)	0 (0.0)	
Normal, ≥80 mL/min	101 (51.0)	983 (69.8)	18.8; <0.00001
Impairment, <80 mL/min	96 (48.5)	425 (30.2)	—
Mild, ≥50 to <80 mL/min	64 (32.2)	359 (25.5)	−6.8; 0.04123
Moderate, ≥30 to <50 mL/min	31 (15.7)	63 (4.5)	−11.2; <0.00001
Severe, <30 mL/min	1 (0.5)	3 (0.2)	−0.3; 0.44038
Disease type, *n* (%)^c^			
cIAI, *N*	65	741	—
Acute gastric or duodenal perforation	4 (6.2)	67 (9.0)	2.9; 0.43116
Appendiceal perforation or periappendiceal abscess	14 (21.5)	364 (49.1)	27.6; 0.00002
Cholecystitis, including gangrenous	21 (32.3)	120 (16.2)	−16.1; 0.00105
Diverticular disease with perforation or abscess	8 (12.3)	57 (7.7)	−4.6; 0.19036
Traumatic perforation of the intestine	0 (0.0)	12 (1.6)	1.6; 0.30158
Peritonitis	8 (12.3)	66 (8.9)	−3.4; 0.36290
Other intra-abdominal abscess	10 (15.4)	55 (7.4)	−8.0; 0.02388
cUTI, *N*	133	667	—
Pyelonephritis	106 (79.7)	550 (82.5)	2.8; 0.44971
cLUTI	27 (20.3)	117 (17.5)	−2.8; 0.44971
Treatment group, *n* (%)^c^			
cIAI, *N*	65	741	—
Ceftolozane/tazobactam + metronidazole	32 (49.2)	357 (48.2)	−1.1; 0.87072
Meropenem	33 (50.8)	384 (51.8)	1.1; 0.87072
cUTI, *N*	133	667	—
Ceftolozane/tazobactam	67 (50.4)	331 (49.6)	−0.8; 0.87444
Levofloxacin	66 (49.6)	336 (50.4)	0.8; 0.87444

*APACHE II* Acute Physiology and Chronic Health Evaluation II, *BMI* body mass index, *cIAI* complicated intra-abdominal infection, *cLUTI* complicated lower urinary tract infection, *cUTI* complicated urinary tract infection, *MITT* microbiologic intention-to-treat, *mMITT* modified microbiologic intention-to-treat, *SD* standard deviation

^a^Percentage difference calculated for patients with history of diabetes versus those with no history of diabetes

^b^Expressed as a percentage of the patients with or without diabetes in the cIAI population only

^c^Expressed as a percentage of the patients with or without diabetes in the cIAI or cUTI population

Notable differences between patients with and without diabetes included age, weight, race, and comorbidities (Table 2). Significantly more patients with than without diabetes were 65 years of age or older; patients with diabetes were also significantly more likely (57.1%) to weigh ≥75 kg at baseline than those without diabetes (44.5%). In addition, there was a significantly higher proportion of Asian patients in the subgroup with diabetes than in the subgroup without diabetes. As expected, renal impairment was more common in the subgroup with diabetes (48.5%) than in the subgroup without it (30.2%); 15.7% of patients with diabetes had moderate renal impairment compared with 4.5% of patients without diabetes. In cIAI, a significantly higher percentage of patients with diabetes had Acute Physiologic Assessment and Chronic Health Evaluation II scores ≥10 (33.8% vs 17.0% in patients without diabetes), potentially driven by older age and decreased renal function. Additionally, cholecystitis was significantly more common in patients with diabetes; appendiceal infections were significantly more common in patients without diabetes.

A summary of medical history ongoing at baseline is shown in Table 3. As expected, cardiac, endocrine, and eye disorders were reported at significantly higher incidences in the subgroup with diabetes. For cardiac disorders, the major driver of the difference between patients with and without diabetes was coronary artery disorders (17.2% vs 7.6%); for eye disorders, the major driver was diabetic retinopathy (4.5% vs 0%).

**Table 3:** Medical history ongoing at baseline (MITT/cIAI population and mMITT/cUTI population)

System organ class, *n* (%)^a^	Diabetes	No diabetes	Difference^b^;
Preferred term	*n* = 198	*n* = 1408	*P* value
Cardiac disorders	50 (25.3)	177 (12.6)	−12.7; <0.00001
Coronary artery disorders	34 (17.2)	107 (7.6)	−9.6; <0.00001
Heart failures	15 (7.6)	39 (2.8)	−4.8; 0.00045
Endocrine disorders	17 (8.6)	57 (4.0)	−4.5; 0.00436
Hypothyroidism	14 (7.1)	41 (2.9)	−4.2; 0.00260
Eye disorders	14 (7.1)	33 (2.3)	−4.7; 0.00022
Diabetic retinopathy	9 (4.5)	0	−4.5; <0.00001
Hepatobiliary disorders	21 (10.6)	82 (5.8)	−4.8; 0.01014
Hepatic and hepatobiliary disorders	13 (6.6)	30 (2.1)	−4.4; 0.00030
Infections and infestations	55 (27.8)	228 (16.2)	−11.6; 0.00006
Urinary tract infections	35 (17.7)	119 (8.5)	−9.2; 0.00004
Viral infectious disorders	10 (5.1)	25 (1.8)	−3.3; 0.00313
Metabolism and nutrition disorders	198 (100.0)	166 (11.8)	−88.2; <0.00001
Glucose metabolism disorders, including diabetes	198 (100.0)	16 (1.1)	−98.9; <0.00001
Lipid metabolism disorders	31 (15.7)	62 (4.4)	−11.3; <0.00001
Obesity	13 (6.6)	37 (2.6)	−3.9; 0.00282
Musculoskeletal and connective tissue disorders	32 (16.2)	114 (8.1)	−8.1; 0.00022
Joint disorders	23 (11.6)	64 (4.5)	−7.1; 0.00004
Nervous system disorders	34 (17.2)	99 (7.0)	−10.1; <0.00001
Peripheral neuropathies	17 (8.6)	1 (0.1)	−8.5; <0.00001
Psychiatric disorders	22 (11.1)	79 (5.6)	−5.5; 0.00284
Depressive disorders	13 (6.6)	33 (2.3)	−4.2; 0.00086
Renal and urinary disorders	67 (33.8)	245 (17.4)	−16.4; <0.00001
Chronic kidney disease	17 (8.6)	19 (1.3)	−7.2; <0.00001
Diabetic nephropathy	12 (6.1)	0 (0.0)	−6.1; <0.00001
Urolithiases	22 (11.1)	81 (5.8)	−5.4; 0.00397
Respiratory, thoracic, and mediastinal disorders	28 (14.1)	87 (6.2)	−8.0; 0.00005
Bronchospasm and obstruction	17 (8.6)	49 (3.5)	−5.1; 0.0007
Vascular disorders	140 (70.7)	390 (27.7)	−43.0; <0.00001
Hypertension	131 (66.2)	342 (24.3)	−41.9; <0.00001

*cIAI* complicated intra-abdominal infection, *cUTI* complicated urinary tract infection, *MITT* microbiologic intention-to-treat, *mMITT* modified microbiologic intention-to-treat

^a^Only preferred terms with differences in rates between patients with and without diabetes are presented

^b^Percentage difference calculated for patients with history of diabetes compared with those with no history of diabetes

Significantly higher incidences of renal diseases and complications were also associated with diabetes, particularly chronic kidney disease (8.6% in patients with diabetes vs 1.3% in patients without diabetes) and diabetic nephropathy (6.1% vs 0%). The significantly higher incidence of vascular disorders in patients with diabetes (70.7% vs 27.7% in patients without diabetes) was largely driven by the incidence of hypertension (66.2% vs 24.3%). Patients with diabetes also had significantly more ongoing infections and more hepatic, nervous system, and respiratory disorders.

Bacteriology findings across subgroups with and without diabetes were generally similar within each indication (cUTI and cIAI; Table 4). *Escherichia coli* was the most common pathogen in both indications and subpopulations.

**Table 4:** Baseline infecting intra-abdominal pathogens and uropathogens (MITT/cIAI population and mMITT/cUTI population)

Pathogen,^a^ *n* (%)	cIAI		cUTI	
	Diabetes *n* = 65	No diabetes *n* = 741	Diabetes *n* = 133	No diabetes *n* = 667
Gram-negative aerobes	46 (70.8)	613 (82.7)	127 (95.5)	637 (95.5)
Enterobacteriaceae	45 (69.2)	577 (77.9)	126 (94.7)	613 (91.9)
*Escherichia coli*	37 (56.9)	488 (65.9)	99 (74.4)	530 (79.5)
*Klebsiella pneumoniae*	0 (0.0)	70 (9.4)	14 (10.5)	44 (6.6)
*Pseudomonas aeruginosa*	0 (0.0)	68 (9.2)	1 (0.8)	22 (3.3)
Gram-positive aerobes	38 (58.5)	406 (54.8)	6 (4.5)	42 (6.3)
*Enterococcus faecalis*	16 (24.6)	79 (10.7)	4 (3.0)	34 (5.1)
*E. faecium*	0 (0.0)	74 (10.0)	0 (0.0)	5 (0.7)
*Staphylococcus aureus*	0 (0.0)	27 (3.6)	2 (1.5)	4 (0.6)
Gram-negative anaerobes	24 (36.9)	267 (36.0)	0 (0.0)	NA
*Bacteroides* spp	23 (35.4)	228 (30.8)	0 (0.0)	NA
Gram-positive anaerobes	7 (10.8)	92 (12.4)	0 (0.0)	NA

*cIAI* complicated intra-abdominal infection, *cUTI* complicated urinary tract infection, *MITT* microbiologic intention-to-treat, *mMITT* microbiologic modified intention-to-treat, *NA*, not applicable

^a^Patients could have had multiple infecting pathogens at baseline

### Efficacy

**Fig. 1:**
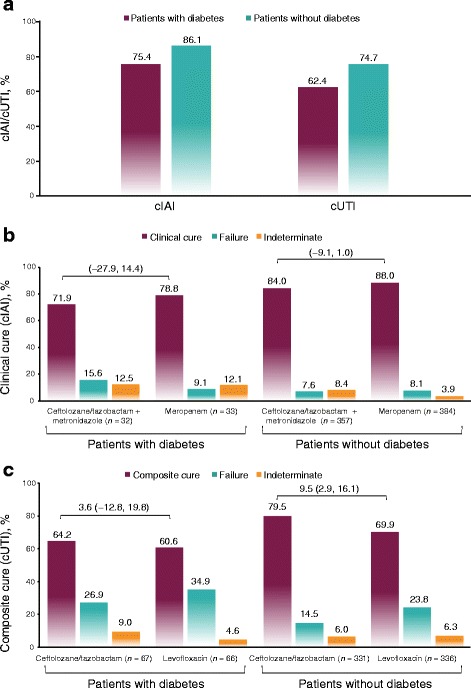
Clinical cure (cIAI) and composite cure (cUTI) in patients with and without diabetes at test-of-cure in cIAI (MITT population) and cUTI (mMITT population) (**a**). Clinical cure at test-of-cure in cIAI (MITT population) (**b**). Composite cure at test-of-cure in cUTI (mMITT population) (**c**). Values above brackets indicate treatment difference (95% confidence intervals)

In general, patients with diabetes had lower cure rates than patients without diabetes (cIAI, 75.4% vs 86.1%, *P* = 0.0196; cUTI, 62.4% vs 74.7%, *P* = 0.1299 [Fig. 1a]). However, cure rates were similar between treatment arms in both indications (Fig. 1b, c), with the exception of significantly higher composite cure rates for ceftolozane/tazobactam than for levofloxacin (79.5% vs 69.9%, *P* = 0.0048) in patients with cUTI but without diabetes (Fig. 1c).

### Safety

Patients with diabetes had significantly higher rates of AEs (49.0% vs 37.3%) and serious AEs (10.6% vs 4.6%) than patients without diabetes (Table 5). However, rates of treatment-related AEs were similar between patients with and without diabetes (8.2% vs 10.1%, respectively), suggesting comorbidities were responsible for differences in AE rates. Types of AEs were generally similar between patient subpopulations, but the incidences of infections and vascular disorders were significantly higher in patients with diabetes.

**Table 5:** Summary of AEs (safety population)

Parameter, *n* (%)	Diabetes *n* = 245	No diabetes *n* = 1802	*P* value^a^
Any AE	120 (49.0)	673 (37.3)	0.00046
Any serious AE	26 (10.6)	82 (4.6)	0.00007
Any treatment-related AE	20 (8.2)	182 (10.1)	0.34038
Any treatment-related serious AE	0 (0.0)	4 (0.2)	0.46052
Any AE leading to discontinuation of study drug	8 (3.3)	32 (1.8)	0.11411
Any treatment-related AE leading to discontinuation of study drug	3 (1.2)	13 (0.7)	0.40162
Any AE resulting in death	6 (2.4)	14 (0.8)	0.01256
Any treatment-related AE resulting in death	0 (0.0)	0 (0.0)	1.00000
System organ class AEs with significant difference between groups			
Infections and infestations	29 (11.8)	137 (7.6)	0.02277
Difference^a^ (95% CI)	−4.2 (−9.0, −0.5)		
Vascular disorders	18 (7.3)	69 (3.8)	0.01046
Difference^a^ (95% CI)	−3.5 (−7.6, −0.7)		

*AE* adverse event, *CI* confidence interval

^a^Calculated for patients with diabetes compared with those without diabetes

## Discussion

In this post hoc analysis of patients with or without a reported medical history of diabetes in the phase 3 ASPECT trials, we have shown that older age, increased weight, and renal impairment are more common in patients with diabetes than in patients without diabetes. In addition, more patients with diabetes had comorbidities and an increased incidence of complicating factors in both cUTI and cIAI, with cardiac, endocrine, and eye disorders reported at significantly higher incidences in the subgroup with diabetes. It has been reported that patients with diabetes are more susceptible to infections and associated complications because of a variety of factors, including but not limited to lower production of interleukins in response to infection and increased virulence of some pathogens in hyperglycemic environments [15].

In our analysis, clinical and composite cure rates were shown to be lower in patients with diabetes but were generally similar between treatment groups, with the exception of significantly higher composite cure rates in ASPECT-cUTI for ceftolozane/tazobactam than for levofloxacin in patients without diabetes. Rates of AEs were also significantly higher in patients with diabetes but were comparable between treatment groups. Overall, the results of this subgroup analysis confirm previous findings in the published literature demonstrating that diabetes increases the risk for poor clinical outcomes and mortality from infectious disease [3, 16–19]. We can postulate that the high levels of complications (including renal and cardiac disorders and additional ongoing infections) in the patient subgroup with diabetes are likely to have had a negative impact on treatment outcomes and that higher rates of AEs in patients with diabetes were also likely due to comorbidities.

This analysis has several limitations, including the post hoc nature of the calculations, which prohibited any statistical significance surrounding the conclusions. Given that the population with diabetes was not prespecified but was defined post hoc based on medical history, the results are contingent on the accuracy of the data reporting and could be confounded by overestimation or underestimation of this patient subgroup. Furthermore, the patient population in the ASPECT studies may not be reflective of the variety of patients seen in clinical practice. Finally, it must be noted that the correlations seen between AE rates, complicating factors, and poorer outcomes among patients with diabetes may be confounded by other unmeasured factors.

## Conclusions

In this post hoc analysis of two phase 3 studies in patients with cIAI and cUTI, baseline factors associated with diabetes included older age, increased weight, and complicating medical factors. Diabetes was associated with lower cure rates and significantly higher AE rates, likely because of the presence of comorbidities. Despite this, ceftolozane/tazobactam was as effective as comparators in treating cUTI and cIAI in patients with diabetes—a population at increased risk for infections and poor clinical outcomes.

## Abbreviations

AE: Adverse event
ASPECT: Assessment of the Safety Profile and Efficacy of Ceftolozane/Tazobactam
cIAI: Complicated intra-abdominal infections
cUTI: Complicated urinary tract infections
IV: Intravenous
q8d: every 8 h
TOC: Test-of-cure
